# Physical and nutrition statuses of geriatric patients after trauma-related hospitalization: Data from the Korean National Health and Nutrition Examination Survey 2013–2015: Erratum

**DOI:** 10.1097/MD.0000000000010217

**Published:** 2018-03-16

In the article, “Physical and nutrition statuses of geriatric patients after trauma-related hospitalization: Data from the Korean National Health and Nutrition Examination Survey 2013–2015”^[1]^, which appeared in Volume 97, Issue 9 of *Medicine*, the conflict of interest should have appeared as:

The statistical consultation was supported by a grant (HI14C1062) from the Korea Health Technology R&D Project through the Korea Health Industry Development Institute (KHIDI), which is funded by the Ministry of Health & Welfare, Republic of Korea.
